# Draft Genome Sequence of the *Mycobacterium tuberculosis* Strain 43-16836, Belonging to the Indo-Oceanic Lineage, Isolated From Tuberculous Meningitis in Thailand

**DOI:** 10.1128/genomeA.00801-13

**Published:** 2013-10-03

**Authors:** W. Viratyosin, S. Kulawonganunchai, N. Smittipat, T. Juthayothin, P. Penpassakarn, E. Pasomsub, W. Chantratita, A. Chaiprasert, P. Palittapongarnpim

**Affiliations:** National Center for Genetic Engineering and Biotechnology, Pathumthani, Thailanda; Department of Microbiology, Faculty of Science, Mahidol University, Bangkok, Thailandb; Department of Pathology, Faculty of Medicine, Ramathibodi Hospital, Bangkok, Thailandc; Department of Microbiology, Faculty of Medicine Siriraj Hospital, Mahidol University, Bangkok, Thailandd

## Abstract

We present the draft genome sequence of *Mycobacterium tuberculosis* strain 43-16836, belonging to the Indo-Oceanic lineage, isolated from a tuberculous meningitis patient in Thailand. The genome is 4,381,942 bp long with 4,316 protein-coding genes and contains new single nucleotide polymorphisms (SNPs), including SNPs of genes that may encode cell wall components and possibly influence virulence.

## GENOME ANNOUNCEMENT

Tuberculous meningitis (TBM), caused by *Mycobacterium tuberculosis*, is the most severe form of extrapulmonary tuberculosis and has the highest rates of mortality and morbidity (1). In Thailand, a molecular epidemiology study has shown that the Beijing (East-Asian), Indo-Oceanic (previously identified and named as Nonthaburi [2]), and Euro-American lineages are the major strains causing TBM (3).

*M. tuberculosis* strain 43-16836 was isolated from the cerebrospinal fluid of a TBM patient in Thailand (4). Importantly, the immunological studies of this strain showed significantly higher levels of tumor necrosis factor and matrix metalloproteinase-9 production during the early infection period of patients within initial stage I (4). The unfavorable clinical outcome of the disease may be the result of the strain-dependent virulence present in this isolate. This isolate has an IS*6110*-restriction fragment length polymorphism (RFLP) pattern belonging to the Nonthaburi family, which a prevalent genotypic cluster in Thailand (2). Recently, subsequent studies based on large sequence polymorphisms and spoligotyping classified this isolate as being of the Indo-Oceanic lineage (lineage I) (5). As the differences in infection outcome may be associated with the genotype, lineage, and sublineage of *Mycobacterium* (6), we performed whole-genome sequencing and analysis to investigate the genetic diversity, phylogeny, and possible strain-dependent virulence features of *M. tuberculosis* 43-16836.

Whole-genome shotgun (WGS) sequencing was performed using the combination of the 454 GS Junior Roche pyrosequencing and Ion Torrent technologies (Ion Torrent Systems, Inc.). For the WGS library, a total DNA read obtained from 454 GS Junior (79,725,780 reads) and the sequencings of two runs (54,489,084 and 49,324,955 reads) by Ion torrent technology were generated. All the resulting reads were aligned against the reference *M. tuberculosis* H37Rv strain and assembled using MIRA 3.4 (7). Variant calling was also performed using Varscan 2.2.11 (8). Automatic genome annotation was performed using tRNAscan, RNAmmer, and the RAST annotation server (9).

The draft genome sequence of *M. tuberculosis* 43-16836 consists of 154 contigs (>200 bp) in a scaffold of 4,381,942 bp, with 34-fold coverage. The assembly genome comprises a single chromosome containing 4,316 protein-coding genes, 3 rRNA genes, and 45 tRNA genes, with a G+C content of 65.5%. The analysis of single nucleotide polymorphisms (SNPs) revealed that this strain shares the SNPs belonging to the Indo-Oceanic lineage and is most closely related to strains of the Philippines sublineage (10). One hundred twenty-one new SNPs, including SNPs of putative genes involved in cell wall synthesis, such as those for membrane indolylacetylinositol arabinosyltransferase EmbC, membrane mannosyltransferase, dTDP-Rha:a-d-GlcNAc-diphosphoryl polyprenol, and a-3-l-rhamnosyl transferase WbbL1, were identified. These newly identified SNPs will be studied further for their functional biological effects.

### Nucleotide sequence accession number.

The draft genome sequence of *M. tuberculosis* 43-16836 was deposited at DDBJ/EMBL/GenBank under the accession no. ATNF00000000. The version described in this study is the first version.
